# Metal-Associated Particulate Matter (PM_2.5_) Induces Cognitive Dysfunction: *Polygonum multiflorum* Improves Neuroinflammation and Synaptic Function

**DOI:** 10.3390/ijms27010230

**Published:** 2025-12-25

**Authors:** Hye Ji Choi, Hyo Lim Lee, Ho Jin Heo

**Affiliations:** Division of Applied Life Science (BK21), Institute of Agriculture and Life Science, Gyeongsang National University, Jinju 52828, Republic of Korea; hjchoi0820@gnu.ac.kr (H.J.C.); gyfla059@gnu.ac.kr (H.L.L.)

**Keywords:** *Polygonum multiflorum*, fine particulate matter (PM_2.5_), cognitive function, oxidative stress, synaptic function

## Abstract

Fine particulate matter (PM_2.5_), which contains heavy metals such as Al, Fe, Mg, and Mn, among others, induces cognitive dysfunction through oxidative stress, neuroinflammation, and impaired mitochondria. This study evaluated the neuroprotective effects of a 40% ethanol extract of *Polygonum multiflorum* (EPM) on PM_2.5_-induced cognitive dysfunction in a mouse model. Behavioral assessments demonstrated attenuated learning and memory impairment following EPM treatment. Redox homeostasis was restored through increased expression of superoxide dismutase (SOD) and glutathione (GSH) and decreased levels of malondialdehyde (MDA) and mitochondrial reactive oxygen species (mtROS) in the EPM group. Mitochondrial function was attenuated, as indicated by recovery of mitochondrial membrane potential and ATP levels. EPM inhibited neuroinflammation by downregulating the TLR4-MyD88-NF-κB pathway and maintaining blood–brain barrier integrity through the upregulation of tight junction proteins. It modulated neuronal apoptosis through the JNK pathway, reducing the accumulation of amyloid-beta and phosphorylated tau. Synaptic plasticity was preserved through upregulation of BDNF/TrkB signaling and cholinergic neurotransmission via regulation of acetylcholine (ACh), acetylcholinesterase (AChE), and choline acetyltransferase (ChAT). To standardize EPM, high-performance liquid chromatography (HPLC) confirmed the presence of the bioactive compound, tetrahydroxystilbene glucoside (TSG). These findings suggest that EPM may be a promising functional food candidate for mitigating PM_2.5_-related cognitive impairments.

## 1. Introduction

Particulate matter (PM) has emerged as a source of critical environmental health concern worldwide [1]. Among the various forms, fine particulate matter (PM_2.5_) is defined as particles with a diameter less than or equal to 2.5 μm [2]. It is of particular concern because of its ability to penetrate deep into the alveoli, enter the bloodstream, and thus affect systemic organs and tissues [2]. PM_2.5_ is directly transmitted to the brain through the olfactory epithelium and the olfactory nerves during breathing or when PM_2.5_ indirectly reaches the brain through the systemic circulation after entering the bloodstream through the alveoli [3]. During this process, PM_2.5_ traverses the blood–brain barrier (BBB), induces oxidative stress along with neuroinflammatory responses within neural tissue, and impairs cognitive function by compromising neuronal integrity [3]. The toxicological profile of PM_2.5_ encompasses various hazardous constituents, including heavy metals (e.g., Al, Fe, Mg), polycyclic aromatic hydrocarbons (PAHs), and organic carbon compounds [2]. In our previous study, we analyzed the inorganic compounds of PM_2.5_ and confirmed that it contains elements such as Al, Fe, Mg, Mn, Ba, Zn, and Cu [4]. In particular, reactive oxygen species (ROS) are continuously generated through redox cycling when various deleterious components, such as transition metals (e.g., Fe, Mn, Cu) and PAHs, which produce hydroxyl radicals and quinones through the Fenton reaction and cytochrome-dependent metabolic processes [5]. PM_2.5_ also activates NADPH oxidase (NOX2) in alveolar macrophages and microglia to promote the production of ROS and inflammatory cytokines [5]. At the same time, PM_2.5_ impairs mitochondrial function to cause electron leakage, reduce ATP synthesis, and lose membrane potential [5]. This exacerbates oxidative stress [5]. Such molecular insults disrupt the balance of the brain’s antioxidant defense system and elevate the levels of inflammatory cytokines, ultimately resulting in sustained oxidative stress, chronic neuroinflammation, and progressive neuronal damage that underlie cognitive impairment [6].

Cognitive impairment is primarily associated with the deterioration in synaptic function, which involves neuroplasticity and cholinergic neurotransmission [7,8]. Neuroplasticity, a process fundamental to memory formation and retention, refers to the capacity of the brain to adapt by adjusting the synaptic strength in response to external stimuli or learning experiences [7]. Brain-derived neurotrophic factor (BDNF) is a key regulator of this process, which activates the tropomyosin receptor kinase B (TrkB) and the transcription factor cAMP response element-binding protein (CREB) [9,10]. The TrkB and the CREB promote neuronal survival and synaptic reinforcement [9,10]. Besides its role in synaptic plasticity, the BDNF–TrkB signaling cascade also engages downstream phosphatidylinositol 3-kinase (PI3K)/protein kinase B (Akt) and extracellular signal-regulated kinase (ERK) 1/2 pathways to contribute to anti-inflammatory and cell survival responses [11]. Importantly, the BDNF/TrkB/CREB signaling axis and the cholinergic system synergistically interact to regulate synaptic plasticity and cognitive function [12]. Acetylcholine (ACh) signaling stimulates BDNF expression through the activation of the muscarinic receptor, and BDNF reciprocally promotes cholinergic neuron function and survival [13]. This bidirectional functional feedback between the two systems is essential for maintaining learning and memory capabilities [13]. However, exposure to PM_2.5_ can reduce neurotransmitters, including acetylcholine, which may impair cognitive function by inhibiting cholinergic signaling [12]. Synaptic plasticity and cognitive performance are further compromised when PM_2.5_-induced oxidative stress and neuroinflammation suppress the expression of BDNF and the signaling of TrkB [12]. Through impaired neuroplasticity, exposure to PM_2.5_ leads to cognitive dysfunctions, including negative effects on learning and memory maintenance [8,10].

*Polygonum multiflorum* (*P. multiflorum*), widely known as ‘He Shou Wu’, belongs to the *Polygonaceae* family and is a medicinal plant traditionally cultivated in East Asian regions, including Korea [14]. This plant has been reported to be effective in preventing hyperlipidemia, aging, inflammation, and age-related cognitive decline [15,16,17]. *P. multiflorum* is rich in bioactive compounds such as 2,3,5,4′-tetrahydroxystilbene-2-O-β-D-glycoside (TSG), anthraquinones including emodin, and various flavonoids and polyphenolic compounds [15,16,17]. In particular, TSG, abundant in *P. multiflorum*, demonstrates antioxidant and anti-inflammatory effects [15,16,17,18]. Moreover, it is recognized for its regulatory influence on essential signaling pathways implicated in cell survival and metabolic processes [18]. Basic research demonstrated that polysaccharides derived from *P. multiflorum* increased the activity of antioxidant enzymes including catalase (CAT), glutathione (GSH), and superoxide dismutase (SOD) in D-galactose-induced aging mice models [15]. The increased activity of the antioxidant enzymes exhibited anti-aging effects through the regulation of the p53/p21 signaling pathway [15]. Lipopolysaccharide (LPS)-induced BV2 cells treated with *P. multiflorum* extract demonstrated anti-inflammatory effects by suppressing the production of inflammatory cytokines, such as tumor necrosis factor (TNF)-α, interleukin (IL)-1β, and IL-6, and modulating mitogen-activated protein kinase (MAPK) signaling pathways [16]. Furthermore, *P. multiflorum* administered in a mouse model was reported to have a protective effect against Aβ_25–35_-induced cognitive decline [17]. The above studies suggest that *P. multiflorum* has tremendous potential as a natural material with protective effects against oxidative stress, inflammation, and neurodegenerative changes particularly attracting attention for its central nervous system (CNS) protective properties. Although extensive research has characterized the effects of metal-associated PM_2.5_ on the respiratory system, few studies have examined the effects of exposure to PM_2.5_ on cognitive impairment. There have been limited experimental investigations addressing underlying mechanisms and potential neuroprotective interventions. Building on previous studies, our research aims to evaluate whether the intake of a root extract of *P. multiflorum* can ameliorate cognitive decline induced by exposure to metal-containing PM_2.5_. Furthermore, a high-performance liquid chromatography (HPLC)–photodiode array detector (PDA) analytical method for quantifying TSG constituents was intended to be developed and validated for expanding industrial applications.

## 2. Results

### 2.1. Analytical Validation of TSG Quantification in EPM

**Figure 1:**
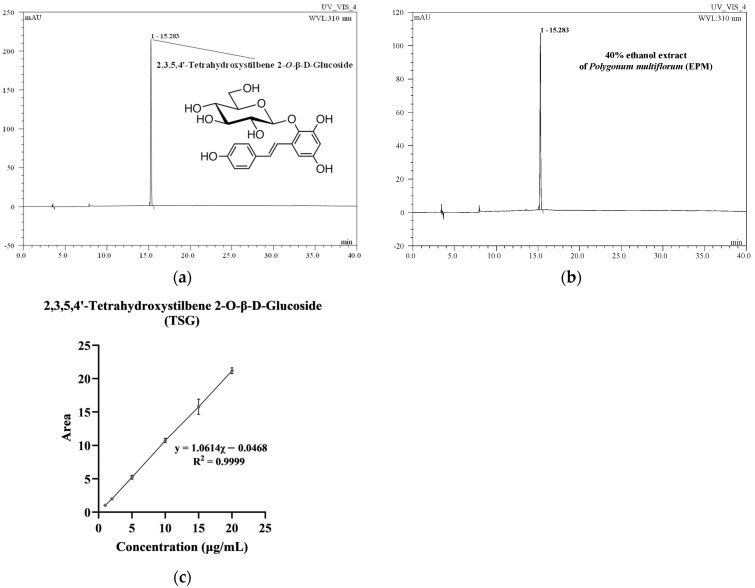
Chromatograms of 2,3,5,4′-tetrahydroxystilbene-2-O-β-D-glycoside (TSG) standard (**a**) and 40% ethanol extract of *Polygonum multiflorum* (EPM) (**b**) recorded by high-performance liquid chromatography (HPLC)–photodiode array detection (PDA) at 310 nm. The calibration curves of TSG (**c**).

HPLC analysis was conducted to determine the content of TSG in the 40% ethanol extract of *P. multiflorum* (EPM). As depicted in Figure 1a,b, both EPM and the TSG standard exhibited chromatographic peaks at a retention time of 15.28 min. The peak observed in the EPM sample at this retention time demonstrated a high degree of similarity to the TSG standard, with a similarity index of 0.999, indicating excellent correspondence between the two compounds. Quantitative analysis was performed using a calibration curve established from the linear correlation between the peak area and the concentration of the TSG standard (Figure 1c). As a result, the TSG content in EPM was calculated to be 93.62 ± 5.82 μg/mg (dried weight), confirming a substantial presence of TSG in the extract.

The calibration curve for TSG was established by plotting the peak area against the corresponding concentrations, yielding a regression equation of y = 1.0614x − 0.0468 with a correlation coefficient (R^2^) of 0.999, indicating excellent linearity within the concentration range of 1–20 μg/mL (Table 1). The limit of detection (LOD) and limit of quantification (LOQ) were 0.32 ± 0.01 μg/mL and 0.97 ± 0.02 μg/mL, respectively, demonstrating the high sensitivity of the analytical method. Accuracy was evaluated through recovery tests at 80%, 100%, and 120% spike levels of TSG content, corresponding to concentrations of 14.41, 18.00, and 21.60 μg/mL, respectively. Recovery rates were 105.32 ± 3.46%, 101.96 ± 0.45%, and 109.13 ± 9.22%, confirming the high accuracy of the analytical method. Precision was assessed regarding repeatability and reproducibility, yielding 3.30% and 1.82% relative standard deviation (RSD) values, respectively. These values fall well below the Association of Official Agricultural Chemists (AOAC)-recommended threshold of 7.3%, indicating excellent precision.

### 2.2. Effect of EPM on PM_2.5_-Induced Cognitive Impairment in Mice

**Figure 2:**
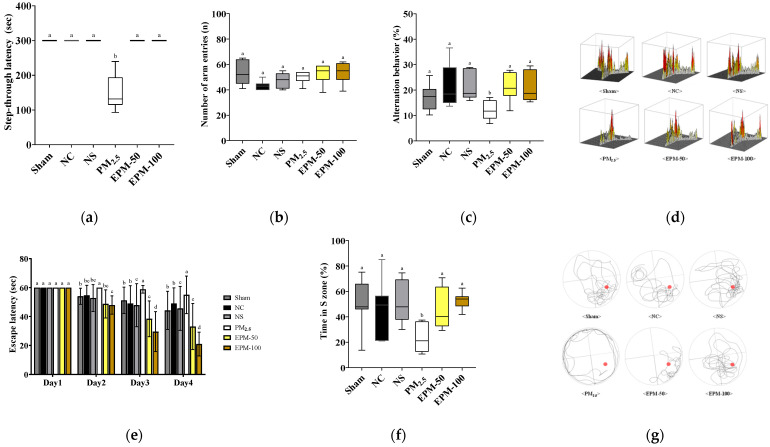
Effect of EPM on cognitive function in PM_2.5_-exposed mice. (**a**) Step-through latency in the passive avoidance test; (**b**) number of arm entries, (**c**) spontaneous alternation behavior, and (**d**) three-dimensional occupancy plots in the Y-maze test, illustrating the spatial distribution of time spent, with bar height and color intensity representing relative dwell time; (**e**) escape latency, (**f**) time spent in the S zone, and (**g**) swimming path images in the Morris water maze test. The red dot indicates the former location of the hidden escape platform during the training trials. All data were expressed as the mean ± standard deviation (SD) (*n* = 7). Different lowercase letters indicate statistically significant differences among groups based on Duncan’s multiple range test (*p* < 0.05).

In the retention trial, the step-through latency was significantly lower in the PM_2.5_-exposed group (147.71 s) relative to the normal control (NC) group (300.00 s), suggesting a decline in memory retention. In contrast, both EPM-treated groups (EPM-50 and EPM-100) exhibited significantly prolonged latencies (300.00 s for both groups) relative to the PM_2.5_ group, suggesting improved memory function (Figure 2a).

During the Y-maze test, the total number of arm entries did not differ significantly between groups, suggesting that the treatments did not impact general locomotor activity. In contrast, spontaneous alternation behavior, an indicator of spatial working memory, was significantly decreased in the PM_2.5_ group (11.84%) compared to the NC group (21.83%). However, both EPM-treated groups (EPM-50 and EPM-100) showed significantly higher alternation percentages (21.59% and 21.65%, respectively) than the PM_2.5_ group, indicating improved working memory performance (Figure 2b–d).

The learning ability of the mice was assessed using hidden platform trials conducted over four consecutive days (Days 1–4). All groups exhibited a gradual reduction in escape latency over the training period. On Day 4, the PM_2.5_ group (55.01 s) showed a significantly longer escape latency compared to the NC group (49.02 s), indicating impaired learning ability. In contrast, the EPM-treated groups (EPM-50 and EPM-100) exhibited significantly shorter latencies (33.07 s and 32.84 s, respectively) relative to the PM_2.5_ group, suggesting improved learning performance. Memory retention was further evaluated using the probe trial by measuring the time spent in the S zone. The PM_2.5_ group (24.99%) spent significantly less time in the S zone compared to the NC group (45.01%), indicating memory impairment. Conversely, the EPM-treated groups (EPM-50: 46.88%; EPM-100: 52.78%) spent significantly more time in the S zone relative to the PM_2.5_ group, indicating enhanced memory retention (Figure 2e–g).

### 2.3. Effect of EPM on PM_2.5_-Induced Oxidative Stress in Brain Tissue

**Figure 3:**
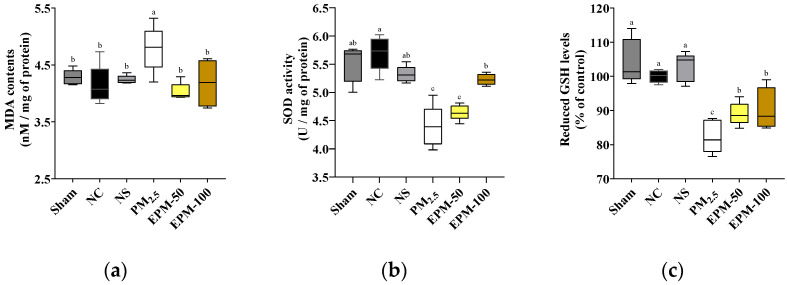
Effect of EPM on the alleviation of oxidative stress in the brain tissues of PM_2.5_-exposed mice. (**a**) MDA content, (**b**) SOD activity, and (**c**) reduced GSH levels. All data were expressed as the mean ± SD (*n* = 5). Different lowercase letters indicate statistically significant differences among groups based on Duncan’s multiple range test (*p* < 0.05).

Malondialdehyde (MDA) content, a key biomarker of lipid peroxidation and oxidative damage, was significantly elevated in the PM_2.5_ group (4.78 nM/mg of protein) relative to the NC group (4.15 nM/mg of protein), indicating enhanced oxidative stress in brain tissue following PM_2.5_ exposure. This elevation was significantly attenuated in EPM-50 (4.03 nM/mg of protein) and EPM-100 (4.18 nM/mg of protein) treatment groups, suggesting that EPM mitigated PM_2.5_-induced lipid peroxidation (Figure 3a).

SOD activity, a crucial enzymatic defense against ROS, was significantly decreased in the PM_2.5_ group (4.44 U/mg of protein) compared to the NC group (5.66 U/mg of protein), reflecting impaired antioxidant capacity in response to PM_2.5_ exposure. This reduction was markedly alleviated by administration of the EPM extract, as evidenced by the partial recovery of SOD activity in the EPM-50 (4.63 U/mg of protein) and EPM-100 (5.23 U/mg of protein) groups (Figure 3b).

Reduced GSH levels, a major non-enzymatic antioxidant, were significantly depleted in the PM_2.5_ group (82.35%) relative to the NC group (100.00%), indicating oxidative imbalance. This decline was significantly attenuated by EPM treatment, with GSH levels recovering to 89.03% and 90.48% in the EPM-50 and EPM-100 groups, respectively (Figure 3c).

### 2.4. Effect of EPM on the Preservation of Mitochondrial Function in PM_2.5_-Exposed Brain Tissue

**Figure 4:**
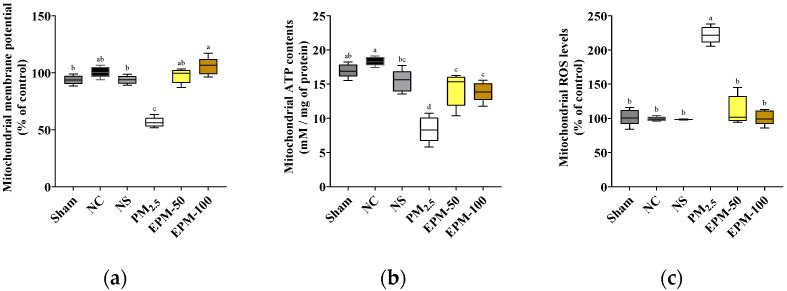
Effect of EPM on the preservation of mitochondrial function in the brain tissues of PM_2.5_-exposed mice. (**a**) Mitochondrial membrane potential, (**b**) mitochondrial ATP contents, and (**c**) mitochondrial ROS levels. All data were expressed as the mean ± SD (*n* = 5). Different lowercase letters indicate statistically significant differences among groups based on Duncan’s multiple range test (*p* < 0.05).

Mitochondrial membrane potential (MMP, ΔΨm) levels were markedly reduced in the PM_2.5_ group (57.11%), indicating substantial mitochondrial dysfunction. This impairment was significantly ameliorated by EPM administration, with MMP levels restored to 96.58% and 106.61% in the EPM-50 and EPM-100 groups, respectively (Figure 4a).

Mitochondrial ATP levels were significantly reduced in the PM_2.5_ group (8.66 nM/mg of protein) compared to the NC group (18.45 nM/mg of protein), indicating compromised mitochondrial bioenergetics. EPM administration effectively restored ATP levels in both treatment groups (EPM-50: 14.00, EPM-100: 13.71 nM/mg of protein), demonstrating significant recovery (Figure 4b).

Mitochondrial ROS (mtROS) levels were markedly elevated in the PM_2.5_ group (224.03%), reflecting exacerbated oxidative stress. This increase was significantly attenuated by EPM administration, with ROS levels reduced to 113.64% and 102.86%in the EPM-50 and EPM-100 groups, respectively (Figure 4c).

### 2.5. Effect of EPM on the Neuroinflammatory Response Induced by PM_2.5_ Exposure

**Figure 5:**
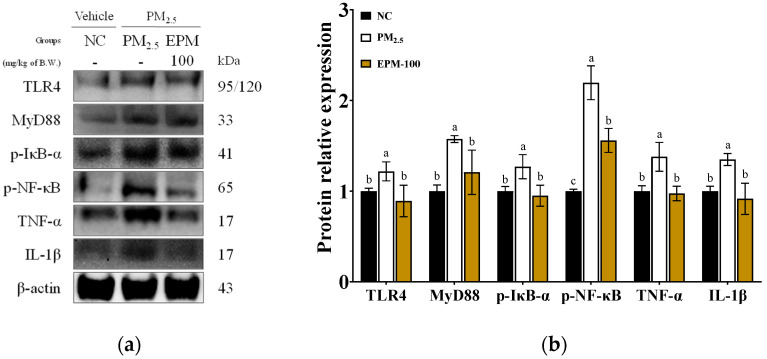
Effect of EPM on the regulation of inflammation in the brain tissues of PM_2.5_-induced mice. (**a**) Western blot images and (**b**) relative expression levels of TLR4, MyD88, p-IκB-α, p-NF-κB, TNF-α, and IL-1β in whole brain tissue (*n* = 3). All data were expressed as the mean ± SD. Different lowercase letters indicate statistically significant differences among groups based on Duncan’s multiple range test (*p* < 0.05).

To assess PM_2.5_-induced neuroinflammation, the expression levels of key inflammatory markers were analyzed in whole brain tissue (Figure 5). The PM_2.5_ group exhibited a significant activation of the toll-like receptor 4 (TLR4) signaling pathway compared to the NC group. The expression levels of TLR4 and its adaptor molecule myeloid differentiation primary response 88 (MyD88) were significantly upregulated to 1.19 and 1.57, respectively. In addition, the NF-κB signaling cascade was activated, as evidenced by increased phosphorylated nuclear factor-κB (p-NF-κB) (2.19) and decreased levels of phosphorylated NF-κB inhibitor α (p-IκB-α) (1.28). Furthermore, the levels of TNF-α and IL-1β were significantly elevated in the PM_2.5_ group, reaching 1.38 and 1.35, respectively. In contrast, treatment with EPM-100 significantly suppressed these inflammatory responses. The expression levels of TLR4 and MyD88 were reduced to 0.87 and 1.21, respectively. p-NF-κB was attenuated to 1.56, while p-IκB-α expression was restored to 0.95. In addition, TNF-α and IL-1β levels were significantly decreased to 0.94 and 0.92, respectively.

### 2.6. Effect of EPM on the Disruption of BBB Integrity Induced by PM_2.5_ Exposure

**Figure 6:**
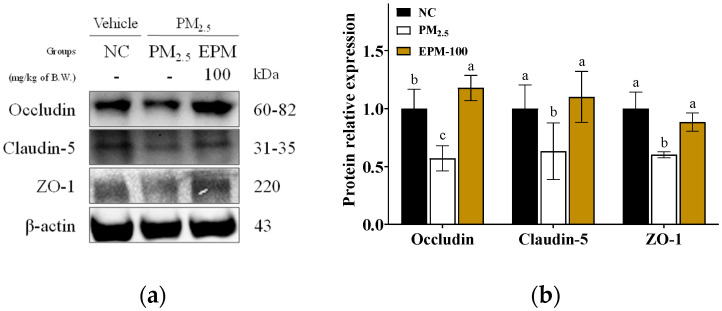
Effect of EPM on the maintenance of BBB integrity in the brain tissues of PM_2.5_-induced mice. (**a**) Western blot images and (**b**) relative expression levels of occludin, claudin-5, and ZO-1 in whole brain tissue. All data were expressed as the mean ± SD. Different lowercase letters indicate statistically significant differences among groups based on Duncan’s multiple range test (*p* < 0.05).

To evaluate PM_2.5_-induced disruption of BBB integrity, the expression levels of tight junction proteins were assessed (Figure 6). The PM_2.5_ group exhibited significant downregulation of occludin, claudin-5, and zonula occludens-1 (ZO-1) compared to the NC group, with relative expression levels of 0.57, 0.63, and 0.60, respectively. In contrast, the EPM-100 group significantly restored the expression levels of these proteins compared to the PM_2.5_ group, with values of 1.20, 1.10, and 0.88, respectively.

### 2.7. Effect of EPM on the Apoptotic Signaling Pathways Induced by PM_2.5_ Exposure

**Figure 7:**
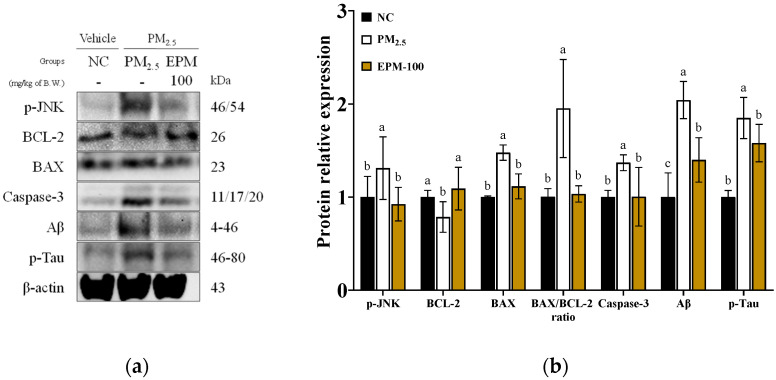
Effect of EPM on the regulation of apoptosis in the brain tissues of PM_2.5_-induced mice. (**a**) Western blot images and (**b**) relative expression levels of p-JNK, BCL-2, BAX, caspase-3, Aβ, and p-tau in whole brain tissue (*n* = 3). All data were expressed as the mean ± SD. Different lowercase letters indicate statistically significant differences among groups based on Duncan’s multiple range test (*p* < 0.05).

To assess PM_2.5_-induced apoptosis in brain tissue, the expression levels of key apoptosis-related markers were evaluated (Figure 7). The PM_2.5_ group exhibited significant upregulation of pro-apoptotic markers compared to the NC group. The expression levels of phosphorylated c-Jun N-terminal kinases (p-JNK), BCL-2-like protein 4 (BAX), BAX/B-cell lymphoma 2 (BCL-2) ratio, caspase-3, amyloid beta (Aβ), and phosphorylated tau (p-tau) were significantly increased to 1.31, 1.48, 1.95, 1.37, 2.04, and 1.85, respectively. In contrast, the anti-apoptotic marker BCL-2 was significantly decreased to 0.79. The EPM-100 group significantly downregulated these apoptotic responses compared to the PM_2.5_ group. The expression levels of pro-apoptotic markers were reduced to 0.92, 1.12, 1.03, 1.00, 1.40, and 1.58, respectively, while BCL-2 expression was restored to 1.09.

### 2.8. Effect of EPM on PM_2.5_ -Induced Synaptic Dysfunction Signaling

**Figure 8:**
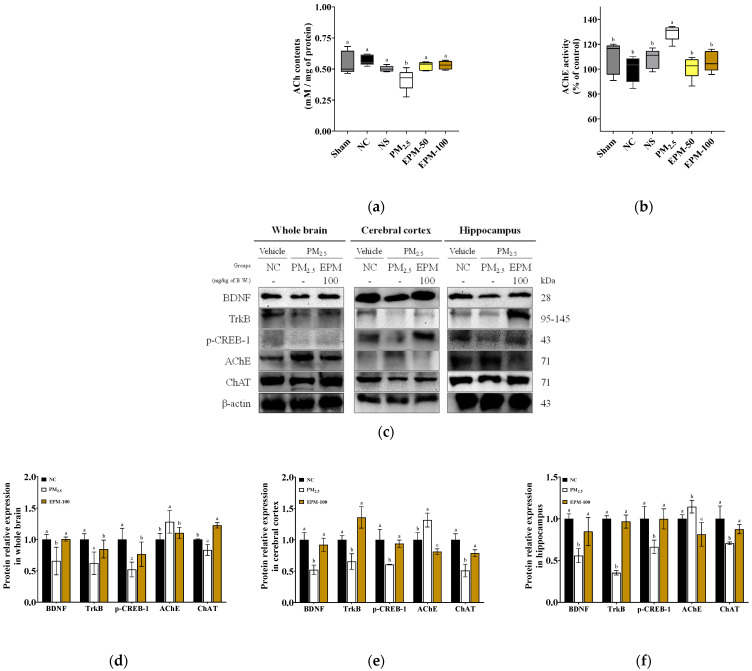
Effect of EPM on synaptic function in the brain tissues of PM_2.5_-exposed mice. (**a**) ACh content and (**b**) AChE activity in whole brain tissue (*n* = 5). (**c**) Western blot images and (**d**–**f**) relative expression levels of BDNF, TrkB, p-CREB-1, AChE, and ChAT in whole brain, cerebral cortex, and hippocampus tissue (*n* = 3). All data were expressed as the mean ± SD. Different lowercase letters indicate statistically significant differences among groups based on Duncan’s multiple range test (*p* < 0.05).

ACh contents were significantly reduced in the PM_2.5_ group (0.41 mM/mg of protein) compared to the NC group (0.57 mM/mg of protein), indicating disrupted cholinergic neurotransmission. This reduction was effectively ameliorated by EPM administration, with ACh contents restored to 0.52 and 0.53 mM/mg of protein in the EPM-50 and EPM-100 groups, respectively (Figure 8a).

Acetylcholinesterase (AChE) activity was significantly elevated in the PM_2.5_ group (129.21%), indicating enhanced degradation of acetylcholine and impaired cholinergic signaling. This elevation was markedly attenuated by EPM treatment, with AChE activity reduced to 101.47% and 106.27% in the EPM-50 and EPM-100 groups, respectively (Figure 8b).

To evaluate synaptic function, cholinergic markers and neuroplasticity-related proteins were measured in the whole brain, cerebral cortex, and hippocampus (Figure 8c–f). The whole brain, cerebral cortex, and hippocampus were selected based on their widely recognized roles in learning and memory, to capture widespread and region-specific alterations in synaptic signaling [19,20]. The PM_2.5_ group significantly disrupted choline acetyltransferase (ChAT) (0.83, 0.51, and 0.71) and increased AChE levels (1.28, 1.32, and 1.14) in the whole brain, cortex, and hippocampus, respectively, compared to the NC group (Figure 8d–f). The EPM-100 group restored cholinergic function, significantly increased expression levels of ChAT (1.23, 0.79, and 0.88), and decreased AChE (1.10, 0.81, and 0.81) levels in the whole brain, cortex, and hippocampus, respectively, compared to the PM_2.5_ group. Moreover, the PM_2.5_ group significantly reduced neuroplasticity markers, as indicated by decreased expression levels of BDNF (0.66, 0.52, and 0.56), TrkB (0.62, 0.65, and 0.35) and phosphorylated CREB (p-CREB)-1 (0.52, 0.61, and 0.66) in the whole brain, cortex, and hippocampus, respectively, compared to the NC group (Figure 8d–f). The EPM-100 group improved these neuroplasticity markers, significantly increased expression levels of BDNF (1.01, 0.92, and 0.85), TrkB (0.85, 1.36, and 0.97), and p-CREB-1 (0.76, 0.94, and 1.00) in the whole brain, cortex, and hippocampus, respectively, compared to the PM_2.5_ group.

### 2.9. Correlation Between Behavioral Performance and Brain Molecular Markers

**Figure 9:**
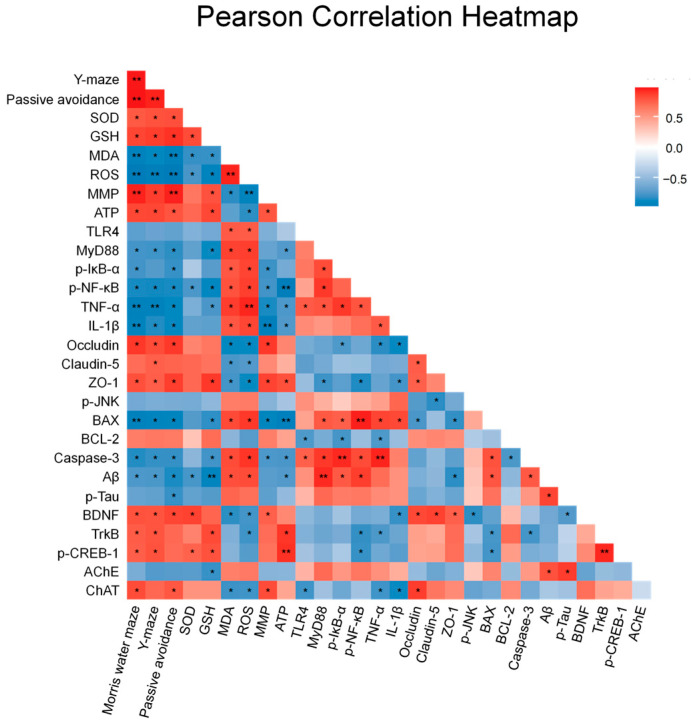
Pearson correlation heatmap between behavioral outcomes and molecular markers in brain tissues of PM_2.5_-exposed mice. The heatmap illustrates the Pearson correlation coefficients between cognitive performance parameters and the protein expression associated with oxidative stress, mitochondrial function, synaptic function, inflammation, apoptosis, and BBB integrity. Color gradients represent the strength and direction of the correlation: red indicates a positive correlation, and blue indicates a negative correlation. Asterisks indicate statistically significant correlations (* *p* < 0.05, ** *p* < 0.01; Benjamini–Hochberg FDR-adjusted for multiple comparisons).

To explore the relationships between behavioral performance and molecular markers, Pearson correlation analysis was conducted and visualized using a heatmap (Figure 9). The results revealed that memory- and learning-related behaviors, including step-through latency in the passive avoidance test, alternation percentage in the Y-maze test, and time spent in the target quadrant of the Morris water maze, were positively correlated with the expression levels of BDNF, TrkB, and p-CREB-1 in whole brain tissue. Additionally, these behavioral parameters negatively correlated with the expression of AChE and pro-apoptotic markers such as BAX and caspase-3. In contrast, a positive correlation was observed between cognitive outcomes and ChAT, MMP, ATP, and SOD levels, suggesting that improved mitochondrial and antioxidant functions are closely linked to cognitive performance. These findings suggest that cognitive impairment induced by PM_2.5_ exposure is associated with dysregulation of neuroplasticity, mitochondrial function, oxidative stress, and apoptosis, which were ameliorated by EPM treatment.

## 3. Discussion

The increasing global prevalence of air pollutants as a public health concern has spurred growing interest in research on related diseases [2,10,21]. Among various air pollutants, PM_2.5_ is known to reach the brain through the olfactory nervous system or systemic circulation and trigger several neuropathological responses, including oxidative stress, neuroinflammation, and impaired neuroplasticity [2,3]. This can ultimately lead to cognitive decline due to neuronal damage and may further progress to neurodegenerative disease [22]. Although economic burden and reduced quality of life are caused by these neurodegenerative diseases that are accompanied by cognitive and physical decline, there is insufficient data on the development of effective treatments and inadequate identification of clear causes of disease [23]. Natural products have attracted attention as promising therapeutic strategies due to their minimal side effects and pharmacological effects that simultaneously modulate various signaling pathways [24]. Reported to have antioxidant and anti-inflammatory properties, the extract of *P. multiflorum* contains various bioactive substances including TSG [25,26]. Therefore, our study aimed to evaluate the neuroprotective effects of EPM to ameliorate cognitive impairment induced by long-term PM_2.5_ exposure.

Clinical studies reported an association between PM_2.5_ exposure and cognitive decline [23,27,28]. In particular, increased PM_2.5_ concentrations have been closely associated with reduced scores on standardized cognitive assessments, such as the Montreal Cognitive Assessment (MoCA) and the Mini-Mental State Examination (MMSE) [21,28]. Increased PM_2.5_ concentrations also lead to increased risk of neurodegenerative diseases such as Alzheimer’s disease [21,28]. Magnetic resonance imaging (MRI) and functional MRI (fMRI) studies have demonstrated that exposure to PM_2.5_ is linked to reduced brain volume and limited connectivity in regions critical for cognitive processing [27,28]. Specifically, the hippocampus, prefrontal cortex, and parietal lobe are affected, and these structural alterations are thought to underlie PM_2.5_-induced cognitive decline [27,28]. Preclinical behavioral experiments have also confirmed that long-term PM_2.5_ exposure negatively affects learning, memory, and attention [4]. To evaluate if cognitive dysfunction was induced by PM_2.5_ exposure, we performed the passive avoidance test, Y-maze test, and Morris water maze test. The behavioral experiments confirmed that long-term exposure to PM_2.5_ induced cognitive dysfunction. Administration of EPM attenuated PM_2.5_-induced cognitive impairment (Figure 2). Consistent with our study findings, a study by Kim [29] found that the administration of *P. multiflorum* extracts improved learning and spatial memory deficits in mice models with scopolamine-induced cognitive impairment, as assessed by passive avoidance, Morris water maze, and novel object recognition tests. Similarly, another study found that the administration of *P. multiflorum* extract in Aβ_25–35_-induced mice significantly improved spatial working memory and long-term memory in passive avoidance and Morris water maze tests [17]. These results suggest that administration of EPM effectively alleviates PM_2.5_-induced impairments of learning memory and recognition memory in mice. Therefore, we performed mechanistic studies to confirm the potential of EPM to alleviate and to identify specific mechanisms associated with PM_2.5_-induced cognitive decline.

PM_2.5_ particles contain redox-active substances, such as heavy metals (e.g., Al, Fe, Mg), PAHs, and LPS, which promote the generation of intracellular ROS [2,24]. In addition, PM_2.5_ contributes to excess ROS production when it inhibits the mitochondrial electron transport chain (ETC) and increases NOX2 activity [30,31]. It also simultaneously depletes endogenous antioxidant defenses and exacerbates oxidative stress [30,31]. Oxidative stress occurs when excess accumulation of intracellular ROS exceeds the defense capacity of antioxidant enzyme systems, such as SOD and GSH, which are responsible for the removal of ROS [24]. Unscavenged ROS oxidize lipids in cell membranes, which leads to altered membrane permeability [5]. It also denatures proteins, impairs enzymatic or receptor function, and damages DNA by inducing mutations or activating cell death pathways [32]. Collectively, these effects cause widespread cellular injury, including that of neurons [33]. In the present study, exposure to PM_2.5_ contributed to redox imbalance and heightened oxidative stress because it was found to not only significantly reduce SOD activity and GSH levels but also increase MDA concentration (Figure 3). In contrast, administration of EPM not only helped to recover SOD and GSH levels but also inhibited the accumulation of MDA. This suggests effective antioxidant activity that attenuates oxidative stress. Our findings are consistent with previous studies utilizing *P. multiflorum* [15,34]. Another study reported that polysaccharides isolated from *P. multiflorum* enhance the ROS scavenging capacity, alleviate oxidative stress, and inhibit neural tissue damage by reducing MDA levels and significantly increasing the activities of key antioxidant enzymes, such as SOD, CAT, and GSH-Px, in the brain tissue of D-galactose-induced aging mice [15]. Through our previous study, we also confirmed that administration of EPM increased SOD and reduced GSH levels in the lung tissue of mice exposed to long-term PM_2.5_, thereby reducing MDA levels and mitigating oxidative stress [34]. Our results support the fact that EPM maintains and activates endogenous antioxidant defense systems to effectively suppress excess oxidative stress. Through this process, EPM positively contributes to neuroprotection and the maintenance of cognitive function.

Oxidative stress causes damage to mitochondria that are central to the regulation of intracellular energy metabolism and ROS homeostasis [20]. Excess ROS damages the mitochondrial inner membrane and cristae structure, inhibits complex I and III functions of the ETC, and increases electron leak and mtROS production [30,35]. The accumulation of mtROS destabilizes the ΔΨm, reduces the efficiency of ATP production, and promotes the opening of the mitochondrial permeability transition pore (mPTP), which ultimately threatens cellular energy metabolism and survival [31]. In our study, mice exposed to PM_2.5_ demonstrated significantly reduced ΔΨm and ATP levels and increased accumulation of mtROS (Figure 4). Our observations demonstrate that PM_2.5_ impairs ETC function and mitochondrial integrity. This impairment leads to disturbances in cellular energy metabolism and ROS homeostasis. In contrast, administration of EPM suppressed the production of mtROS and restored ΔΨm and ATP levels. These results confirmed a partial improvement in impaired mitochondrial function. Consistent with the present results, previous studies reported that TSG protects mitochondrial function in Aβ-induced neurotoxicity models by restoring ΔΨm and suppressing ROS [36]. Moreover, TSG mitigated calcium overload, reduced ROS accumulation, and suppressed mitochondrial toxicity to exhibit tissue-protective effects in a D-galactose-induced ischemia model [37]. A lung-protective effect was observed in a previous study where administration of EPM in a long-term PM_2.5_ exposure model protected mitochondrial function in lung tissue by improving mitochondrial ROS accumulation, enhancing ΔΨm, and increasing ATP levels [34]. Recently, Wang [38] reported that TSG modulates the AMP-activated protein kinase (AMPK)/Silent information regulator 2 homolog 1 (SIRT1) signaling pathway to activate the mitochondrial ecosystem signaling and to maintain energy activity. Therefore, EPM may aid neuronal cell survival by stabilizing mitochondria and maintaining cellular energy metabolism under PM_2.5_-induced pathological stress.

Along with the accumulation of mtROS, mitochondrial dysfunction induces inflammatory responses at the systemic and the CNS levels [31]. When brain tissue is penetrated by PM_2.5_, which contains constituents such as PAHs and heavy metals (e.g., Al, Fe, Mg), TLR4 expressed on microglial surfaces becomes activated [39]. Activated TLR4 initiates a MyD88-dependent signaling cascade that promotes the degradation of IκB-α [39]. This results in nuclear translocation of NF-κB and subsequent upregulation of pro-inflammatory cytokines, including IL-1β and TNF-α [39]. Activation of the TLR4/MyD88/NF-κB pathway is a principal mechanism underlying excess microglial inflammatory responses that contribute to neuronal damage and the development of chronic neuroinflammation [22]. Our study observed an increase in the activation of the TLR4/NF-κB pathway and in the expression of pro-inflammatory cytokines in the brains of PM_2.5_-induced mice (Figure 5). In contrast, administration of EPM suppressed the activation of the TLR4/NF-κB pathway and the expression of pro-inflammatory cytokines to ameliorate PM_2.5_-induced neuroinflammatory responses. Consistent with our findings, Zhang [26] reported that TSG inhibited the TLR4–NF-κB pathway in LPS-stimulated BV2 microglia. The inhibited TLR4–NF-κB pathway collectively attenuated the overall inflammatory by suppressing the expression of TNF-α, IL-1β, iNOS [26]. It also suppressed the production of NO and ROS as well as inhibited NOX2 activity [26]. Additionally, Jiao [40] demonstrated that TSG modulates the inflammatory response of microglia by suppressing Aβ_1–42_-induced secretion of pro-inflammatory cytokines, such as TNF-α, IL-1β, and IL-6 from microglia, and promoting factors associated with tissue repair and anti-inflammatory functions, including IL-10 and BDNF. TSG also regulates the expression of the transcription factor purine-rich box-1 (PU.1), a key regulator of microglial differentiation and polarization, thereby contributing to the overall attenuation of neuroinflammation [40]. These findings suggest that EPM, which is abundant with TSG, may protect cognitive function by alleviating neuroinflammation induced by PM_2.5_ exposure.

Persistent inflammatory responses also affect the structural stability of the CNS [3]. Inflammatory cytokines act on vascular endothelial cells to inhibit the expression of tight junction (TJ) proteins and to impair the selective permeability of the BBB [3]. The role of the BBB is to maintain the homeostasis of the CNS by restricting the movement of molecules between the bloodstream and brain parenchyma [41]. Hence, it blocks the entry of pathological proteins (Aβ, p-tau) and pro-inflammatory factors [41]. An association between impaired BBB integrity and cognitive decline has been reported in various neurodegenerative diseases, which acts as a pathophysiological mechanism that mediates the penetration of pathological proteins and inflammatory factors into the brain parenchyma [41,42]. TJ proteins are essential to maintain the structural stability and barrier function of the BBB [41,42]. Claudin-5 and occludin seal the gap between endothelial cells and ZO-1 connects them to the cytoskeleton to stabilize the structure of the junctional complex [41,42]. In the present study, we observed the expression of occludin, claudin-5, and ZO-1 was significantly reduced in the PM_2.5_-exposed group (Figure 6). In contrast, the administration of EPM restored the expression of the TJ proteins and partially preserved BBB function. A previous study also demonstrated that TSG enhances barrier function by upregulating ZO-1 and occludin expression in a dextran sulfate sodium (DSS)-induced colitis model [43]. In a high-fat diet model, *P. multiflorum* extracts were found to alleviate endothelial oxidative stress and the levels of expressed tight junction proteins [44]. These findings support the direct involvement of TSG in endothelial protection and in the maintenance of TJ integrity. Our results suggest that EPM suppresses PM_2.5_-induced neuroinflammation to maintain the expression of tight junction proteins at the BBB level.

When the integrity of the BBB is disrupted, pathological proteins and inflammatory mediators penetrate the CNS and create a toxic environment that directly affects neuronal survival [3,42]. This environment induces intracellular oxidative stress and inflammation [25]. Inflammatory cytokines, such as TNF-α and IL-1β, and excessive ROS act as central mechanisms that induce intrinsic apoptosis by mediating phosphorylation of the JNK pathway within the MAPK signaling pathway [25]. Phosphorylated JNK activates the transcription factor c-Jun to increase the expression of BAX and inhibits the expression of the anti-apoptotic protein BCL-2, thereby increasing the BAX/BCL-2 ratio [45]. This dysregulation induces the collapse of the ΔΨm and the opening of the permeability transition space, which mediates the cascade of activation of caspase-9 and caspase-3 through the release of cytochrome c [36]. In our study, we observed increased expression of p-JNK, increased BAX/BCL-2 ratio, and upregulation of caspase-3 in the PM_2.5_ exposure group, which suggested the activation of mitochondria-dependent apoptosis (Figure 7). We also noted significant accumulation of pathological proteins (Aβ and p-tau) in Alzheimer’s disease, which was consistent with established Alzheimer’s pathology [8,20,46]. Accelerated apoptosis and impaired synaptic function result from the accumulation of these pathological proteins, which are key pathogenic mechanisms to induce cognitive decline [8,46]. In our study, we noted that the accumulation of Aβ and p-tau was significantly reduced and the apoptotic proteins were downregulated when EPM was administered. Our results demonstrate that EPM contributes to neuronal survival by regulating intrinsic apoptotic pathways. In addition, EPM reduced the accumulation of pathological proteins, thereby supporting the maintenance of synaptic function. Consistent with our findings, Kim [29] reported that the mitochondrial pathway was modulated when *P. multiflorum* extract inhibited apoptosis by decreasing BAX and increasing BCL-2 expression in the brains of scopolamine-induced cognitively impaired mice models. Similarly, Jiao [36] reported that neurogenesis and cognitive recovery were promoted when TSG, a constituent of *P. multiflorum*, reduced the expression of pro-apoptotic proteins BAX and caspase-3, while increasing the expression of anti-apoptotic BCL-2 in an Aβ-induced hippocampal neuronal injury model. These results suggest that TSG-rich EPM may alleviate cognitive decline by suppressing neuronal apoptosis and the accumulation of pathological proteins under pathologically stressed environments.

Cognitive impairment is a result of the main pathological mechanisms, apoptosis and accumulation of pathological proteins, which damage synaptic structure and function [8,20]. These changes are induced by both chronic increases in inflammatory cytokines as well as the accumulation of Aβ and p-tau, which inhibit synaptic protein expression and decrease the efficiency of neurotransmission, especially in the hippocampus and cerebral cortex [12,46]. Synapses provide the physiological basis for neurotransmission and play a crucial role in adapting to external stimuli and long-term memory within the brain [7]. Synaptic plasticity refers to changes in synaptic efficacy in response to increased or decreased synaptic activity over time [47]. This process is essential for learning and memory formation [47]. The BDNF/TrkB signaling pathway plays a crucial role in regulating synaptic plasticity and mediating ChAT expression that supports acetylcholine synthesis and synaptic transmission efficiency in the cholinergic system [13,47]. BDNF interacts with the TrkB receptor and triggers downstream signaling pathways that result in the phosphorylation of CREB, which is a crucial molecular mechanism to regulate synaptic plasticity [9,10]. By promoting long-term memory formation and regulating synaptic gene expression, p-CREB contributes to the stability of synaptic structures, protein synthesis, and long-term potentiation [9]. p-CREB also induces ChAT transcription and supports the functional maintenance of cholinergic neurons [9]. Inhibition of CREB phosphorylation leads to abnormalities in synaptic function and structure, which threaten the survival of cholinergic neurons and can lead to loss of neurons [8,9,10]. The cholinergic system refers to the process by which neurons transmit neural signals using ACh as a neurotransmitter, which maintains synaptic excitability and transmission efficiency [8]. Impaired cholinergic neurotransmission is recognized as a major pathological mechanism that contributes to cognitive decline [8], with alterations in the expression of ChAT and AChE serving as key molecular indicators that reflect reduced synaptic plasticity and behavioral deficits [8]. Therefore, disruption of the BDNF/TrkB/p-CREB pathway impairs both synaptic integrity and cholinergic system function, ultimately leading to deficits in memory and learning [13]. Diminished efficiency of synaptic signaling and subsequent deficits in memory and learning abilities are due to impaired acetylcholine neurotransmission caused by decreased ChAT expression and increased AChE activity in the hippocampus and cerebral cortex [48]. In our study, PM_2.5_ exposure significantly decreased the expression of BDNF, TrkB, p-CREB-1, and ChAT, and significantly increased the expression of AChE in the whole brain, cortex, and hippocampus (Figure 8). In contrast, administration of EPM ameliorated PM_2.5_-induced synaptic dysregulation and improved the expression of the associated proteins. Similarly, Chen [47] reported that chronic administration of TSG prevented synaptic loss and cognitive impairment by increasing the expression of BDNF and CREB, maintaining synaptic plasticity in the hippocampus, and enhancing memory function. Likewise, TSG, the major stibene compound of *P. multiflorum*, was reported to increase the levels of ACh and to inhibit the activity of AChE in a scopolamine-induced memory impairment mouse model, resulting in improved cholinergic function and memory performance [29]. The BDNF/TrkB pathway has been reported to regulate synaptic function and to control inflammatory responses and apoptosis through NF-κB inhibition and JNK blockade [9,11]. In our study, administration of EPM was shown to restore the BDNF/TrkB signaling, inhibit the NF-κB and JNK pathways, normalize the BCL-2/BAX ratio, and reduce caspase-3 activity. Pearson correlation analysis in our study revealed positive correlations between behavioral measures of cognitive function and levels of BDNF, TrkB, p-CREB-1, ChAT, while AChE activity showed a negative correlation with markers of oxidative stress and inflammation (Figure 9). Costa [49] reported a positive correlation between BDNF signaling and cognitive function. Our findings suggest that the metal-containing PM_2.5_-induced synaptic dysregulation and cognitive impairment may occur through the BDNF/TrkB/p-CREB signaling pathway and the cholinergic system. We also demonstrate that the neuroprotective effects of EPM may be firmly associated with the restoration of these pathways. However, the lack of investigator blinding and the absence of a positive control represent limitations of this study. To strengthen the current findings, subsequent research should employ blinded experimental designs and include a positive control group to provide a more comprehensive comparison. Despite these limitations, our findings suggest that EPM may have neuroprotective potential, supporting further investigation in additional preclinical studies.

## 4. Materials and Methods

### 4.1. Sample Preparation

In this study, dried *P. multiflorum* root was purchased from Donguiherb (Seoul, Republic of Korea), originally cultivated in Seosan, Chungcheongnam-do, Republic of Korea. The dried roots were extracted with 40% ethanol at 40 °C for 2 h. The extract was filtered using No. 2 filter paper, concentrated using a rotary evaporator, and then lyophilized. The lyophilized extract was stored at −18 °C until used. Before administration, it was dissolved in drinking water for oral administration to mice.

### 4.2. HPLC Analysis and Method Validation

#### 4.2.1. HPLC Conditions

HPLC analysis was performed following the method described in a previous study [13]. An Ultimate 3000 HPLC system (Dionex, Sunnyvale, CA, USA) equipped with a YMC-Triart C_18_ column (250 × 4.6 mm, 5 μm; YMC Korea, Seongnam, Republic of Korea) was used. Freeze-dried 40% ethanol extract of *P*. *multiflorum* (EPM) and the reference compound 2,3,5,4′-tetrahydroxystilbene-2-O-β-D-glucoside (TSG; Tokyo Chemical Industry Co., Tokyo, Japan) were dissolved in 50% methanol, filtered through a 0.45 μm syringe filter, and 20 μL of each sample was injected. The mobile phases consisted of solvent A (0.1% formic acid in distilled water) and solvent B (0.1% formic acid in acetonitrile), with a 1 mL/min flow rate. The PDA detection wavelength was set at 310 nm.

#### 4.2.2. Method Validation

The HPLC method developed for quantifying TSG in the extract was validated for linearity, LOD, LOQ, accuracy, and precision. Method validation parameters were established based on the AOAC Analytical Laboratory Accreditation Criteria Committee (ALACC) guide, “How to Meet ISO 17025 Requirements for Methods Verification” [50].

Linearity was assessed by analyzing TSG standard solutions at concentrations of 1, 2, 5, 10, 15, and 20 μg/mL, each measured in triplicate. The calibration curve was generated by plotting peak area versus concentration.

The LOD and LOQ for the analytical method were determined based on the slope (S) of the calibration curve and the standard deviation (σ) of the y-intercept, as defined by the following equations:

LOD = 3.3 × (σ/S)(1)

LOQ = 10 × (σ/S)(2)

The σ represents the standard deviation of the y-intercept derived from the calibration curve regression, and S denotes the slope of the calibration curve. Calibration curves were established using a minimum of five standard solutions, with triplicate measurements performed at each concentration level. The parameters S and σ were obtained through linear regression analysis of the replicate data.

Recovery experiments were conducted to assess the method accuracy. TSG standard solutions were prepared at concentrations of 14.41, 18.00, and 21.60 μg/mL, corresponding to 80%, 100%, and 120% of the TSG content in the EPM. Each standard solution was mixed in equal volume with the EPM (200 μg/mL). The mixtures were then analyzed by HPLC, and recovery rates were calculated based on the detected TSG levels.

Recovery (%) = ((observed mixture concentration-actual EPM concentration) ÷ actual TSG concentration) × 100(3)

Precision was assessed in terms of repeatability and reproducibility. Repeatability was evaluated by analyzing the linearity of samples over three consecutive days. Reproducibility was assessed by measuring the TSG standard solutions at 1, 2, 5, 10, 15, and 20 μg/mL five times each. Precision was expressed as the relative standard deviation (RSD %).

RSD % = standard deviation/mean × 100(4)

### 4.3. Animals and Treatments

The experimental protocol was approved by the Institutional Animal Care and Use Committee (IACUC) of Gyeongsang National University (approval number: GNU-230303-M0039, approval date: 3 March 2023). All the procedures followed ethical guidelines to minimize animal pain, stress, and discomfort. Six-week-old male BALB/c mice were purchased from Samtako Bio (Osan, Republic of Korea) and randomly housed as five mice per cage. The animals were allowed an acclimatization period of one-week before the experiments. Mice were maintained under controlled environmental conditions with a 12 h light/dark cycle, temperature of 22 ± 2 °C, and relative humidity of 50 ± 5%, with free access to food and water throughout the study.

The animals were randomly divided into six groups: sham control (Sham; without chamber exposure), normal control (NC; clean air exposure), normal sample (NS; clean air exposure + 100 mg/kg body weight of EPM), negative control (PM_2.5_ exposure only), low-dose sample (EPM-50; PM_2.5_ exposure + 50 mg/kg body weight of EPM), and high-dose sample (EPM-100; PM_2.5_ exposure + 100 mg/kg body weight of EPM). Using the RAND() function in Excel, mice were randomly allocated to groups to maintain objectivity in group assignment. The sequences of treatments and cage arrangements were randomly assigned and rotated regularly to mitigate biases related to order and position. The NC and PM_2.5_ groups were orally administered drinking water, while the NS, EPM-50, and EPM-100 groups received EPM once daily, 1 h before each chamber exposure, throughout the PM_2.5_ exposure period without any pretreatment. Based on HPLC quantification, EPM contained 93.6 μg/mg of TSG. Accordingly, oral administration of 50 and 100 mg/kg EPM corresponded to approximately 4.68 mg/kg and 9.36 mg/kg TSG, respectively. These doses are below the hepatotoxic dose ranges reported for purified TSG in rodent studies [51]. Each group consisted of 20 mice that were pre-assigned to independent experimental analyses prior to study initiation, including behavioral testing (*n* = 7), antioxidant and cholinergic system assays (*n* = 5), mitochondrial function analysis (*n* = 5), and Western blot analysis (*n* = 3).

### 4.4. Chamber Exposure Condition

Details on the whole-body PM_2.5_ exposure system have been previously described by [4]. The PM_2.5_ utilized in this experiment was commercially obtained as Arizona test dust (0–3 µm; Powder Technology Inc., Arden Hills, MN, USA) and suspended in distilled water before use. An inorganic compound analysis confirmed the presence of Al, Mg, Ba, Zn, Fe, Cu, and Pb [4]. According to the World Health Organization (WHO) global air quality guidelines (AQG), the recommended 24 h average exposure level for PM_2.5_ is less than 25 µg/m^3^ [1]. In our study, mice in the PM_2.5_, EPM-50, and EPM-100 groups were exposed to aerosolized PM_2.5_ at a concentration of 500 µg/m^3^ for 5 h per day, 5 days per week, for 12 weeks. This concentration was selected based on previously established experimental conditions and relevant literature [4,24]. As controls, filtered air was provided to the NC and NS groups.

### 4.5. Behavioral Test

#### 4.5.1. Passive Avoidance Test

The passive avoidance apparatus consisted of a light chamber and a dark chamber connected by a door. On the first training day, each mouse was placed in the light chamber with the door closed and allowed to habituate for 1 min, followed by an additional 2 min exposure under illumination. The door was then opened, and the latency to enter the dark chamber time taken for all four paws to cross into the dark chamber was measured and recorded. Once the mouse had fully entered the dark chamber, the door was immediately closed, and an electric foot shock was delivered through the floor grid at an intensity of 0.5 mA for 3 s. After training for 24 h, the mice underwent the same habituation procedure, and latency to enter the dark chamber was measured again. A maximum latency of 300 s was set as the cut-off time.

#### 4.5.2. Y-Maze Test

The Y-maze consisted of three arms (33 × 15 × 10 cm each) constructed from acrylic. Mice were placed on the same arm, and their movements were recorded for 8 min using a Smart video tracking system (version 3.0, Panlab, Barcelona, Spain). Spontaneous alternation behavior was defined as successive entries into three different arms without repetition, forming overlapping triplet sequences.

#### 4.5.3. Morris Water Maze Test

The Morris water maze consisted of a circular pool with a diameter of 90 cm and a height of 30 cm, filled with water maintained at 22 ± 2 °C. A 10 cm in diameter circular escape platform was placed in one quadrant and submerged 1 cm below the water surface. Behavioral trials were recorded using a Smart video tracking system (version 3.0; Panlab, Barcelona, Spain), with a maximum trial duration of 60 s. Acquisition training was conducted over four consecutive days, with four trials per day. During each trial, mice were released from randomly selected start points and allowed to search for the hidden platform. If a mouse failed to locate the platform within 60 s, it was gently guided to the platform and permitted to remain there for 20 s. On the fifth day, a probe trial was performed in which the platform was removed. The time spent in the S zone, where the platform had previously been located, was recorded for 60 s to assess spatial memory retention.

### 4.6. Tissue Preparation

Following completion of the designated experimental procedures, mice were euthanized via CO_2_ inhalation, and brain tissues were immediately collected for subsequent biochemical analyses. The whole brain was excised from each animal and mechanically minced to enable subdivision of tissue from the same individual for multiple assays. Tissue aliquots obtained from the same brain were processed according to the requirements of each assay. Specifically, whole brain tissues were homogenized in phosphate-buffered saline (PBS, pH 7.4) for SOD, MDA, ACh, and AChE assays, and in phosphate buffer (pH 6.0) for GSH analysis. The homogenates were centrifuged at 2350× *g* for 10 min for MDA, 10,000× *g* for 15 min for GSH, and 13,572× *g* for 30 min for ACh and AChE analyses. The resulting supernatants were used for each respective assay, whereas the pellet obtained after centrifugation at 400× *g* for 10 min was used for the SOD assay. All centrifugation steps were performed at 4 °C. Protein concentrations were quantified using the Bradford assay with the Bio-Rad protein assay dye reagent (Bio-Rad Laboratories Inc., Hercules, CA, USA).

### 4.7. Antioxidant System

#### 4.7.1. MDA Content

The level of MDA was measured using the thiobarbituric acid reactive substances (TBARS) assay. Brain tissue supernatant was mixed with 0.67% thiobarbituric acid (TBA) and 1% phosphoric acid, then heated at 95 °C for 1 h. After heating, the mixture was centrifuged to remove any precipitates, and the absorbance of the resulting supernatant was measured at 532 nm.

#### 4.7.2. SOD Activity

SOD activity was assessed using a commercial SOD assay kit (Dojindo Molecular Technologies, Kumamoto, Japan) by the manufacturer’s instructions.

#### 4.7.3. Reduced GSH Level

The level of reduced GSH was measured by mixing the brain tissue supernatant with 5% metaphosphoric acid, followed by centrifugation at 2000× *g* for 2 min. The supernatant was reacted with 1 mg/mL o-phthaldialdehyde, 0.26 M Tris-HCl buffer (pH 7.5), and 0.65 N NaOH. Fluorescence intensity was measured using the fluorometer (Infinite F200, Tecan, Männedorf, Switzerland) at excitation and emission wavelengths of 360 nm and 430 nm, respectively.

### 4.8. Mitochondrial Function

#### 4.8.1. Mitochondrial Isolation

Mitochondria were isolated from whole brain tissue according to a previously described method [24]. The protein concentration of the mitochondrial fraction was determined using the Bradford assay with the Bio-Rad protein assay dye reagent (Bio-Rad Laboratories, Hercules, CA, USA).

#### 4.8.2. Mitochondrial Membrane Potential (ΔΨm)

For ΔΨm measurement, isolated mitochondria were incubated in respiration buffer supplemented with 5 mM pyruvate and 5 mM malate as respiratory substrates. 5,5′,6,6′-tetrachloro-1,1′,3,3′-tetraethylbenzimidazolyl carbocyanine iodide (JC-1) was added at a final concentration of 1 μM and incubated at 37 °C for 15 min in the dark. JC-1 fluorescence was measured using a fluorometer with excitation at 530 nm and emission at 590 nm for J-aggregates (indicative of high ΔΨm, red fluorescence) and at 530 nm for monomers (indicative of low ΔΨm, green fluorescence).

#### 4.8.3. ATP Content

ATP content in isolated mitochondria was determined using a commercial bioluminescence assay kit (ENLITEN^®^ ATP Assay System; Promega Corp., Madison, WI, USA), following the manufacturer’s instructions.

#### 4.8.4. Mitochondrial ROS (mtROS)

Mitochondrial ROS production was assessed using the fluorescent probe 2′,7′-dichlorofluorescein diacetate (DCF-DA). Isolated mitochondria were incubated at 37 °C in respiration buffer containing 125 mM KCl, 2 mM KH_2_PO_4_, 2.5 mM malate, 20 mM 2-[4-(2-hydroxyethyl)piperazin-1-yl]ethanesulfonic acid (pH 7.4), 1 mM MgCl_2_, 5 mM pyruvate, and 500 μM 2-[4-(2-hydroxyethyl)piperazin-1-yl]ethanesulfonic acid. DCF-DA was added at a final concentration of 25 μM, and the mixture was incubated for 30 min in the dark. ROS-induced fluorescence was measured using a fluorometer with excitation at 485 nm and emission at 535 nm.

### 4.9. Cholinergic System

#### 4.9.1. ACh Content

ACh content was measured using the alkaline hydroxylamine colorimetric method. 2 M hydroxylamine hydrochloride and 3.5 N NaOH were mixed at a 1:1 (*v*/*v*) ratio to prepare the alkaline hydroxylamine reagent. The sample was then mixed with the reagent at room temperature for 1 min. A color-developing solution containing 0.5 N HCl and 0.3 M iron (III) chloride hexahydrate was subsequently added in equal volume. The absorbance of the resulting color complex was measured at 540 nm using a microplate reader (Epoch 2; BioTek Instruments Inc., Winooski, VT, USA).

#### 4.9.2. AChE Activity

AChE activity was measured using Ellman’s colorimetric method. The sample was incubated with 50 mM sodium phosphate buffer (pH 7.4) at 37 °C for 15 min. Following incubation, the mixture was reacted with Ellman’s reagent at 37 °C for 10 min. The absorbance of the resulting yellow-colored product was measured at 405 nm using a microplate reader (Epoch 2; BioTek Instruments Inc., Winooski, VT, USA).

### 4.10. Western Blot

Western blot analysis was performed on previously published methods [24] using mouse brain tissues. Details of the antibodies used in this study are summarized in Table 2. Uncropped Western blot membranes are provided in the Supplementary Material (Figures S1–S4).

### 4.11. Correlation and Visualization Analysis

Pearson correlation analysis was conducted to assess the relationships between behavioral parameters and the expression levels of molecular markers. A heatmap was generated from the resulting correlation matrix using R software (version 4.4.2, R Foundation for Statistical Computing, Vienna, Austria) to represent the strength and direction of the correlations visually. Color gradients were applied to indicate correlation direction and magnitude, with red representing positive correlations and blue representing negative correlations. Only correlations with FDR-adjusted *p* < 0.05 were marked with asterisks (* *p* < 0.05, ** *p* < 0.01) in the heatmap.

### 4.12. Statistical Analysis

All experimental data were expressed as the mean ± standard deviation. Statistical analyses were performed using SAS software (version 9.4; SAS Institute Inc., Cary, NC, USA) and GraphPad Prism (version 10.0, GraphPad Software, Boston, MA, USA). The normality of data distribution was assessed using Shapiro–Wilk test. One-way analysis of variance (ANOVA) was used to evaluate differences among groups, followed by Duncan’s multiple range test for post hoc comparisons. Statistical significance was set at *p* < 0.05.

To control for false discovery rate arising from multiple comparisons across biochemical endpoints and correlation analyses, all *p*-values were adjusted using the Benjamini–Hochberg (BH) false discovery rate correction method. Statistical significance was determined based on FDR-adjusted *p*-values, with significance levels defined as * *p* < 0.05 and ** *p* < 0.01.

## 5. Conclusions

In conclusion, our study confirmed that oral administration of EPM attenuated PM_2.5_-induced cognitive impairment and associated neuropathological changes through multiple biological mechanisms. EPM enhanced endogenous antioxidant activity and alleviated oxidative stress, as evidenced by the increased GSH levels and SOD activity. Furthermore, EPM protected mitochondrial function by restoring the mitochondrial membrane potential, elevating the production of ATP, and reducing mtROS levels. The TLR4–MyD88–NF-κB signaling pathway was inhibited by EPM with the suppression of neuroinflammatory responses. Notably, the integrity of the BBB was preserved, as indicated by the upregulated expression of tight junction proteins. There was reduced accumulation of Aβ and p-tau proteins because EPM modulated neuronal apoptosis by regulating the JNK pathway. High ACh contents and ChAT levels and reduced AChE activity indicated that EPM enhanced synaptic plasticity through increased BDNF/TrkB signaling along with improved cholinergic neurotransmission. Both the BDNF/TrkB signaling pathway and the cholinergic neurotransmission system are involved in maintaining synaptic plasticity. Dysregulation in these systems may negatively impact a wide range of cognitive functions, including learning and memory. EPM treatment enhanced these critical neurobiological mechanisms that subsequently translated into significant improvements in cognitive function, as demonstrated by enhanced performance in the Morris water maze, passive avoidance, and Y-maze behavioral tests. Our study enhances our understanding of metal-associated PM_2.5_ neurotoxicity mechanisms and suggests therapeutic potential for natural products. In addition, by validating the method to detect TSG, a major bioactive component in EPM, we provide a foundation for future industrial applications.

## Figures and Tables

**Table 1:** Validation parameters of HPLC-PDA analysis for 2,3,5,4′-Tetrahydroxystilbene-2-O-β-D-glycoside (TSG).

Parameters	2,3,5,4′-Tetrahydroxystilbene-2-O-β-D-glycoside (TSG)		
Linearity range (μg/mL)	1–20		
Regression equation	y = 1.0614x − 0.0468		
Correlation coefficient (R^2^)	0.999		
Intra-day precision (RSD %)	3.30		
Inter-day precision (RSD %)	1.82		
LOD (μg/mL)	0.32 ± 0.01		
LOQ (μg/mL)	0.97 ± 0.02		
Recovery rate (%)	80%	100%	120%
	105.32 ± 3.46	101.96 ± 0.45	109.13 ± 9.22

**Table 2:** Primary and secondary antibody information, as used in this study.

Antibody	Catalog NO.	Concentration	Manufacturer
β-Actin	sc-69879	1:1000	Santa Cruz Biotechnology (Dallas, TX, USA)
TLR4	sc-293072	1:1000	
MyD88	sc-74532	1:1000	
p-IκB-α	sc-8404	1:1000	
p-NF-κB	sc-136548	1:1000	
TNF-α	sc-33639	1:1000	
IL-1β	sc-515598	1:1000	
Occludin	sc-133256	1:1000	
Claudin-5	sc-374221	1:1000	
ZO-1	sc-33725	1:1000	
p-JNK	sc-6254	1:1000	
BCL-2	sc-7382	1:1000	
BAX	sc-7480	1:1000	
Caspase-3	sc-56053	1:1000	
Aβ	sc-28365	1:1000	
p-Tau	sc-32275	1:1000	
TrkB	sc-377218	1:1000	
p-CREB-1	sc-81486	1:1000	
AChE	sc-373901	1:1000	
BDNF	#47808	1:1000	Cell Signaling Tech (Rosemont, IL, USA)
ChAT	#27269	1:1000	
Goat-anti-rabbit IgG	#7074	1:5000	
Goat-anti-mouse IgG	#1724044	1:5000	Bio-Rad (Richmond, CA, USA)

## Data Availability

The original contributions presented in this study are included in the article. Further inquiries can be directed to the corresponding author.

## Supplementary Materials

The following supporting information can be downloaded at https://www.mdpi.com/article/10.3390/ijms27010230/s1.
