# Supplementary material for: Metal-Associated Particulate Matter (PM2.5) Induces Cognitive Dysfunction: Polygonum multiflorum Improves Neuroinflammation and Synaptic Function
Source: Int J Mol Sci. 2025 Dec 25;27(1):230. doi: 10.3390/ijms27010230 (PMC12785887; doi:10.3390/ijms27010230)
Supplement: Supplementary file 1 [file ijms-27-00230-s001.zip › ijms-4027098-supplementary.pdf]

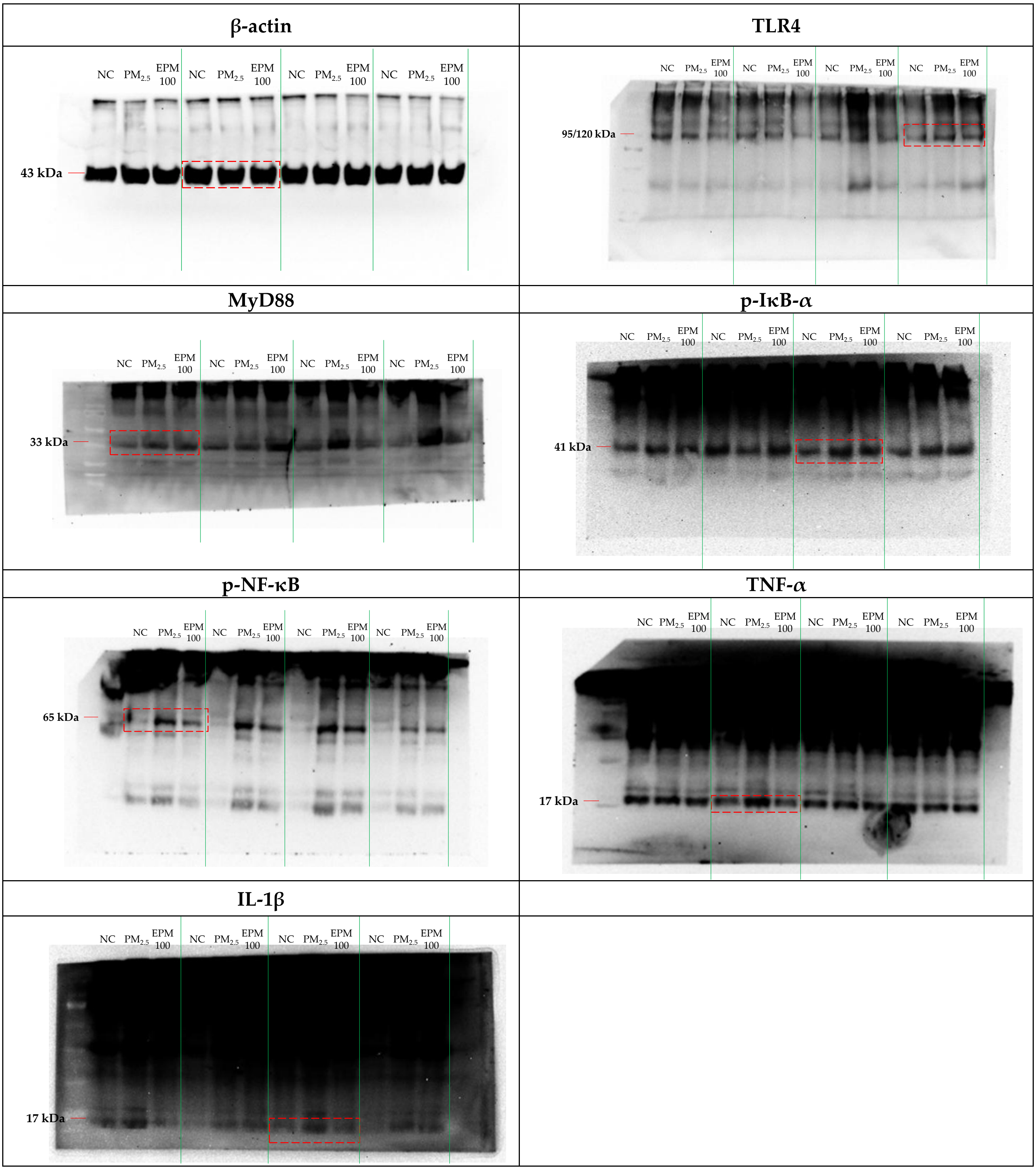

Supplementary Figure S1. Uncropped, full-length Western blot membranes corresponding to the representative blots shown in Figure 5. Membranes were probed for Toll-like receptor 4 (TLR4), myeloid differentiation primary response 88 (MyD88), phosphorylated IκB-α (p-IκB-α), phosphorylated NF-κB (p-NF-κB), tumor necrosis factor-α (TNF-α), interleukin-1β (IL-1β), and β-actin. These full-length membranes were used for densitometric quantification, and representative cropped images are presented in the main figure. One sample was excluded from densitometric analysis due to technical quality criteria.

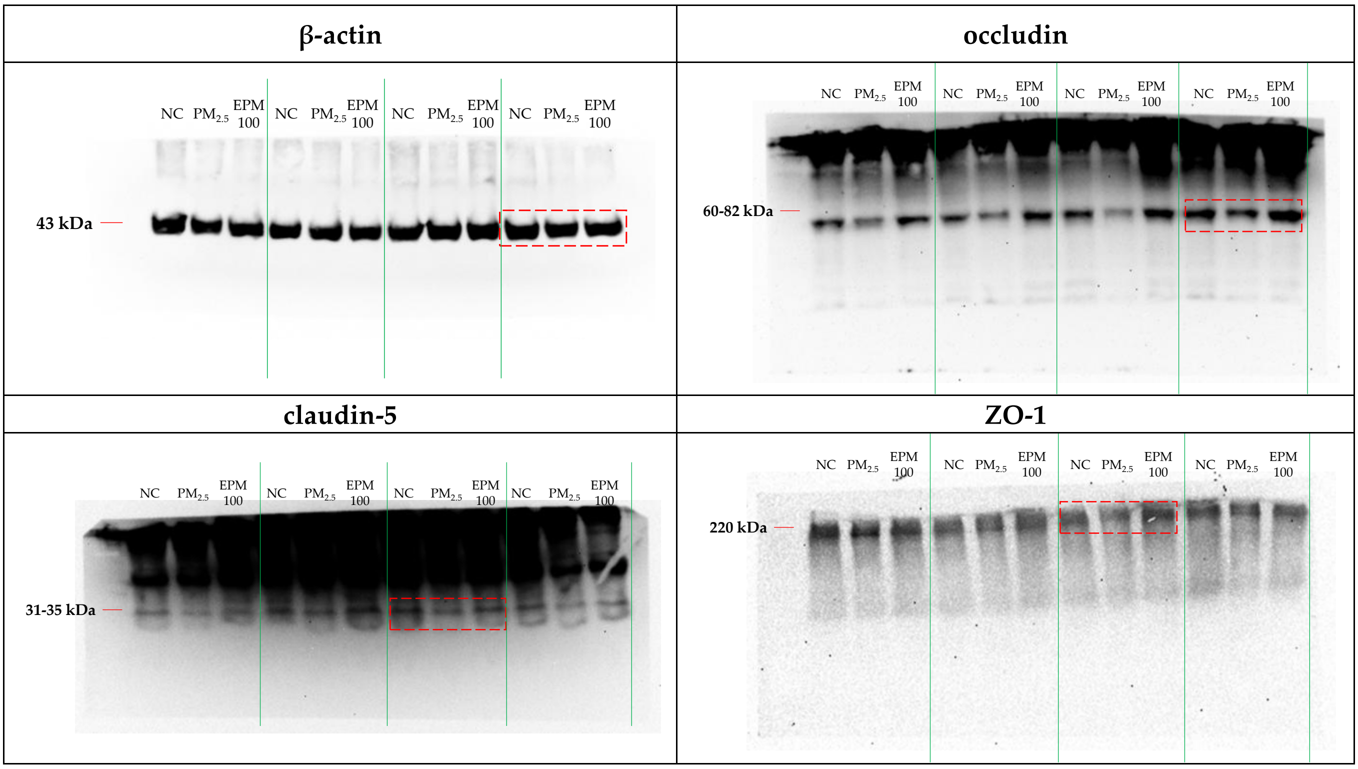

Supplementary Figure S2. Uncropped, full-length Western blot membranes corresponding to the representative blots shown in Figure 6. Membranes were probed for occludin, claudin-5, zonula occludens-1 (ZO-1), and β-actin. These full-length membranes were used for densitometric quantification, and representative cropped images are presented in the main figure. One sample was excluded from densitometric analysis due to technical quality criteria.

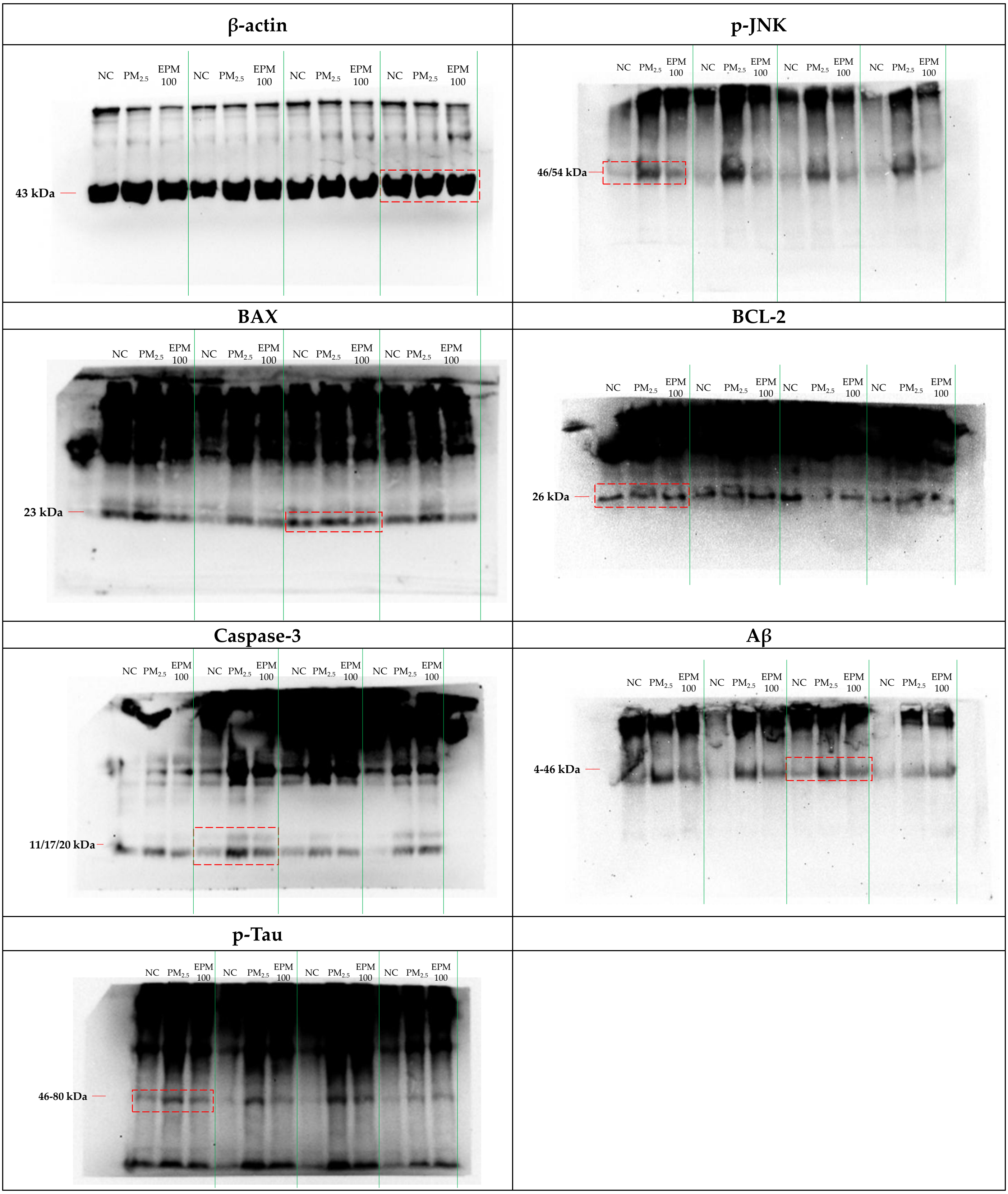

Supplementary Figure S3. Uncropped, full-length Western blot membranes corresponding to the representative blots shown in Figure 7. Membranes were probed for phosphorylated c-Jun N-terminal kinase (p-JNK), B-cell lymphoma 2 (BCL-2), BCL-2-associated X protein (BAX), caspase-3, amyloid-β (Aβ), phosphorylated tau (p-tau), and β-actin. These full-length membranes were used for densitometric quantification, and representative cropped images are presented in the main figure. One sample was excluded from densitometric analysis due to technical quality criteria.

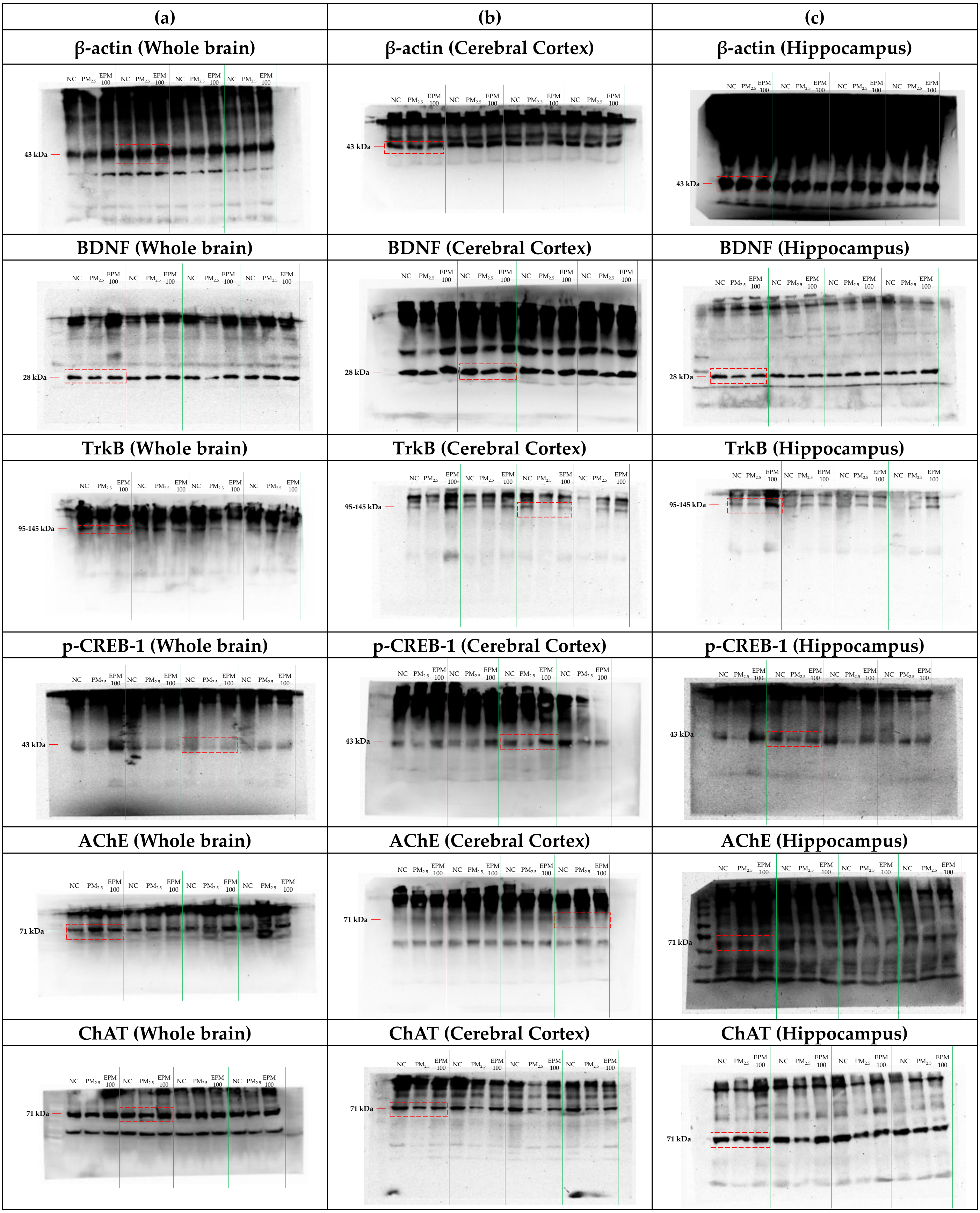

Supplementary Figure S4. Uncropped, full-length Western blot membranes corresponding to the representative blots shown in Figure 8. Membranes were probed for brain-derived neurotrophic factor (BDNF), tropomyosin receptor kinase B (TrkB), phosphorylated cAMP response element-binding protein-1 (p-CREB-1), acetylcholinesterase (AChE), choline acetyltransferase (ChAT), and β-actin. Panels are shown by tissue type: (**a**) whole brain, (**b**) cerebral cortex, and (**c**) hippocampus. These full-length membranes were used for densitometric quantification, and representative cropped images are presented in the main figure. One sample was excluded from densitometric analysis due to technical quality criteria.
